# CeDaD—a novel assay for simultaneous tracking of cell death and division in a single population

**DOI:** 10.1038/s41420-025-02370-7

**Published:** 2025-03-04

**Authors:** Lukas Nöltner, Kurt Engeland, Robin Kohler

**Affiliations:** Molecular Oncology, Faculty of Medicine, University of Leipzig, Leipzig, Germany

**Keywords:** Cell division, Cell death

## Abstract

The cell division cycle and the various forms of programmed cell death are interconnected. A prominent example is the tumor suppressor p53, which not only induces apoptosis but also plays an important role in the arrest of the cell cycle. Consequently, simultaneous analysis of cell division and cell death is frequently of significant interest in cell biology research. Traditionally, these processes require distinct assays, making concurrent analysis challenging. To address this, we present a novel combined assay, called *CeDaD* assay—*Cell Death and Division* assay—which allows for the simultaneous quantification of cell division and cell death within a single-cell population. This assay utilizes a straightforward flow cytometric approach, combining a staining based on carboxyfluorescein succinimidyl ester (CFSE) to monitor cell division with an annexin V-derived staining to assess the extent of cell death.

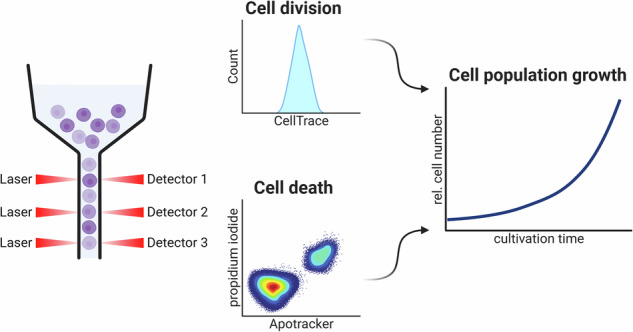

## Introduction

In cell biology research, the analysis of various aspects of cellular physiology—including cell signaling, cell–cell interactions, differentiation, as well as the examination of disease models and the impact of drugs and compounds—is typically conducted using cell culture models. Two fundamental processes that define the growth dynamics of a cell culture population are cell division and cell death, whose dysfunctional interplay is central to the development of cancer.

Cell division, the outcome of the cell cycle, whereby a single cell divides into two daughter cells, is the primary process driving the expansion of a cell population and is subject to tight regulatory control [1, 2]. Cell cycle regulation is largely mediated through protein phosphorylation and transcriptional control [3–5].

Cell death, in contrast, contributes to the reduction of a cell population and can be categorized into necrosis and apoptosis, along with various subtypes and intermediate forms such as necroptosis and pyroptosis. Among these, apoptosis is the primary mechanism responsible for physiological cell death [6–9].

Cell cycle and apoptosis regulation are intricately linked through multiple signaling pathways, amongst which those involving the tumor suppressor p53 are particularly prominent. This protein plays a dual role by inducing apoptosis and causing cell cycle arrest through partially divergent and partially overlapping signaling pathways [10, 11]. A key mechanism by which p53 connects apoptosis induction to cell cycle arrest is through its impact on the RB/E2F and DREAM/MuvB transcriptional systems through indirect inhibition of cyclin-dependent kinases (CDKs). This p53-DREAM/RB pathway leads to the transcriptional repression of genes encoding central cell cycle regulators and ultimately causes cell cycle arrest [12–17]. Consequently, a loss of RB and the DREAM/MuvB component LIN37 leads to a derepression and deregulation of cell cycle genes [18].

Given the central role of cell cycle and apoptosis regulation in both cancer prevention and treatment researchers have sought ways to identify compounds that can effectively target and disrupt these signaling pathways when they malfunction. For example, specific inhibitors have been developed to target the many kinases that control the cell cycle [4]. Only recently, CDK7 was identified as a critical activator of the main CDKs required for cell cycle control and has therefore become a promising new target for cancer therapy [19–21]. Apart from CDKs, several other kinases are essential for a successful cell division. For instance, polo-like kinase 1 (PLK1) is required for various phases of mitosis including mitotic entry and spindle formation [22]. Inhibition of PLK1 by volasertib leads to mitotic arrest and is approved for the treatment of acute myeloid leukemia by the FDA [23]. p53 function has also been a major focus of investigation. One approach involves inhibiting the activity of mouse double minute 2 (MDM2), an E3 ubiquitin ligase that facilitates the proteolysis of p53 [10].

To evaluate the growth rate of a cell population without a detailed insight into cell division activity and cell death rate, numerous assay systems have been developed [24]. The simplest and most direct method for assessing cell population growth is cell counting, which can be performed either via microscopy or in suspension following trypsinization [25]. However, both direct cell counting methods are labor-intensive. To address this, automated microscopic cell counting has been recently developed, particularly utilizing deep learning [26–28]. Alternatively, several chemical high-throughput assays indirectly measure population growth. For instance, the *ATP* assay estimates cell population growth based on ATP concentration, while the tetrazolium salt-based colorimetric *MTT* and *WST* assays measure NADH concentration either directly or indirectly [29–31]. Although these assays are popular due to their simplicity, they are susceptible to artifacts that researchers must be aware of, as they only indirectly infer cell population growth based on metabolic markers [32–35].

In order to obtain quantitative information about cell cycle activity within a cell population, only a few assays are commonly used in addition to qualitative methods that analyze cell cycle phase distribution, such as DNA content staining or *Fluorescent Ubiquitination-based Cell Cycle Indicators* [25]. The most commonly employed assays for quantifying cells undergoing DNA replication within a defined time window are staining using bromodeoxyuridine (BrdU) and staining with its derivative ethynyldeoxyuridine (EdU). However, these assays are prone to artifacts, such as endoreduplication without completing the cell cycle, which can occur due to interference with the mitotic machinery [25, 36, 37]. Alternatively, dye dilution assays, like carboxyfluorescein succinimidyl ester (CFSE) and the second-generation CFSE-derived *CellTrace* staining, measure cell divisions by tracking dye concentration per cell after a defined incubation period. During each cytokinesis, the dye in the mother cell is equally distributed between the daughter cells [38–40]. These stainings can be quantified using live cell flow cytometry.

Cell death, alongside cell division, is a crucial factor in describing the growth of a cell population. Various assays specialize in detecting cell death through different mechanisms [41]. For instance, the *TUNEL* assay visualizes late apoptotic cells undergoing DNA fragmentation, either microscopically or via flow cytometry [42, 43]. Caspase assays identify apoptotic cells by detecting active caspase forms in immunoassays [44, 45]. More recently, label-free approaches to identify cell death have been explored [46]. Finally, one of the most commonly used labeling-based methods, annexin V and propidium iodide (PI) double staining, detects apoptotic and dead cells by assessing the loss of membrane integrity and asymmetry [41, 47]. Since traditional annexin V staining relies on calcium binding, a calcium-independent fluorogenic peptide alternative, known commercially as *Apotracker Green*, has been developed to detect apoptotic cells [48].

To our knowledge, live cell imaging, with or without additional dyes, is the only method available to directly analyze both cell division and cell death within a single-cell population. However, this approach is often limited by technical complexity, image quality issues, cell motility, and morphological changes [49–54]. Consequently, despite the availability of methods to separately analyze cell cycle activity and cell death, the ability to quantify both processes simultaneously remains highly limited.

Here, we present an easy-to-use flow cytometric assay, named *CeDaD* assay—*Cell Death and Division* assay—for simultaneous analysis of cell cycle activity and cell death within a single-cell population. This novel assay combines two well-established, commercially available assays: the CFSE-based *CellTrace Violet* assay and annexin V-based *Apotracker Green* staining with PI. This allows for rapid assessment of both cell division and cell death in small samples. In addition, simple calculations can generate exponential growth curves.

To validate the *CeDaD* assay, we analyzed a colorectal carcinoma cell line treated with three compounds targeting p53 and cell cycle pathways—AMG 232 (MDM2 inhibitor), YKL-5-124 (CDK7 inhibitor), and volasertib (PLK1 inhibitor) [23, 55–57].

## Results and discussion

### Cell count and *WST* assay show an impact of three compounds on cell population growth, but cannot differentiate between cell cycle arrest and cell death induction

**Fig. 1:**
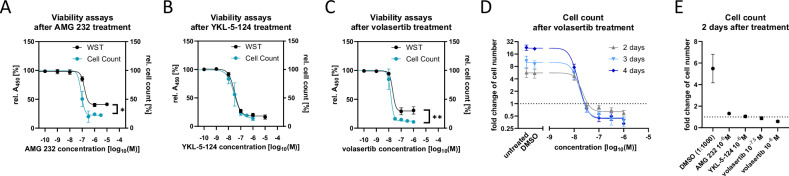
Cell count and WST assay indicate an impact of three compounds on cell population growth, but cannot differentiate between cell cycle arrest and cell death induction. HCT116 wt cells were treated with varying concentrations of AMG 232 (**A**), YKL-5-124 (**B**), or volasertib (**C**) for 2 days. Relative absorbance (A_450_) in *WST* assay and relative cell count by cell counting compared in relation to the DMSO control (*n* ≥3). The concentration-dependent effect of volasertib on cell count was compared after 2, 3, and 4 days of volasertib treatment (**D**, *n* ≥3). Cell counting 2 days after treatment with drug concentrations that were used for further tests was compared between treatments (**E**, *n* ≥3). Mean ± SD are given, and *P* values were calculated by two-way ANOVA (*P <0.05; **P <0.01; ***P <0.001). Concentration-response curves were calculated by least square regression with variable hill slope.

As model compounds interfering with cell division and viability, the MDM2 inhibitor AMG 232, the CDK7 inhibitor YKL-5-124, and the PLK1 inhibitor volasertib were utilized [23, 55, 56, 58–61]. Although the antiproliferative effects of these compounds are well-documented, their impact on HCT116 cells was validated using *WST* assay and cell counting (Fig. 1). Both methods revealed a strong, concentration-dependent reduction in HCT116 cell growth after 48 h of treatment (Fig. 1A–C). Interestingly, while YKL-5-124 produced consistent results across both assays (Fig. 1B), significant discrepancies were observed in effect sizes for AMG 232 and volasertib between cell counting and *WST* assays (Fig. 1A, C). Notably, only direct cell counting allows for the accurate measurement of fold changes in cell number relative to the initial population. Furthermore, prolonged treatment times revealed additive inhibitory effects, with 10^−^^6^ M volasertib causing a reduction in total cell number by day 2. By day 3 and 4, the increased effect size was attributed primarily to enhanced control growth rather than a drop in the minimum cell number (Fig. 1D). For later evaluation of the *CeDaD* assay, drug concentrations with significant impact on cell population growth were selected (Fig. 1E).

Our data highlight the limitations of metabolic assays, such as the *WST* assay, compared to the more direct method of cell counting. While these assays can be valuable high-throughput tools, their potential artifacts, masked by low standard deviations, must be actively considered. Without such caution, they can create a false sense of accuracy. The discrepancies observed with AMG 232 and volasertib treatments illustrate this point, emphasizing the need for cross-validation with methods such as cell counting. Consequently, cell counting was preferred over the *WST* assay for comparison with the *CeDaD* assay.

### Cell division and cell death rates can be analyzed from one cell sample

The *CeDaD* assay combines *CellTrace Violet*, PI, and *Apotracker Green* staining, with absorption and emission spectra optimized for simultaneous flow cytometric analysis. This method is designed to track cell division and cell death events within a single population of less than one million cells.

**Fig. 2:**
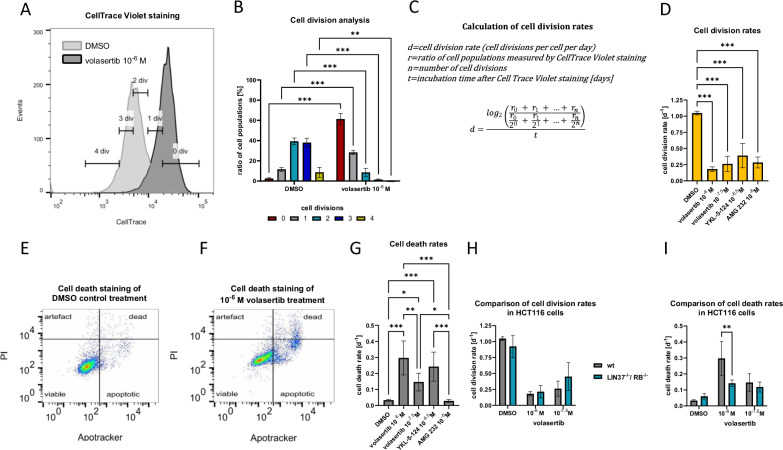
Cell division and cell death rates can be analyzed from one cell sample. HCT116 wt cells were treated for 2 days with AMG 232, YKL-5-124, or volasertib. Representative *CellTrace Violet* staining of DMSO control and 10^−6^ M volasertib-treated cells was gated by number of cell divisions within 2 days (**A**). Cell division distribution was compared between both conditions (**B**, *n* = 3). Based on the cell division analysis and following the appropriate equation (**C**) cell division rates were calculated as average cell divisions per day (**D**). For cell death analysis, cell populations were gated by *Apotracker Green* and propidium iodide (PI) staining. In representative gating analyses of DMSO control (**E**) and 10^−6^ M Volasertib-treated cells (**F**) were gated as viable, apoptotic, dead, or artifact. Cell death rates represented by apoptotic and dead cell populations were compared between treatments (**G**, *n* = 3). Cell division rates (**H**) and cell death rates (**I**) of the DMSO control and after volasertib treatment (10^−6^ M and 10^−7.5^ M) were compared between HCT116 wt and LIN37^−/−^/RB^−/−^ cells. Mean ± SD are given, and *P* values were calculated by two-way ANOVA (*P <0.05; **P <0.01; ***P <0.001).

To analyze cell division activity, single cells were gated into categories of no, one, two, three, or four divisions based on decreasing *CellTrace Violet* staining after 48 h of incubation. Given the short doubling time of HCT116 cells in the literature (16–29 h) and in our cell counting experiments (20.9–21.6 h), up to three divisions are likely and even four divisions are possible [62, 63]. Gating is exemplified for DMSO and 10^−6^ M volasertib-treated cells in Fig. 2A. Treatment with 10^−6^ M volasertib significantly reduced populations undergoing two to four divisions while increasing the proportion of cells dividing once or not at all (Fig. 2B).

The equation used to calculate the cell division rate of a population is depicted in Fig. 2C. This equation assesses the theoretical ratio of cell numbers 2 days after and before *CellTrace Violet* staining, based on the proportion of cells with different division counts. As one cell produces two daughter cells per cell cycle, it assumes that two cells that were gated into the one-cell division population derived from one initial mother cell. Accordingly, four cells within the population after two cell divisions and eight cells from the three cell divisions population each stem from one initial cell. Given that cell division is an exponential process, a logarithm with base two is applied to calculate the average number of divisions per cell over 2 days. To express this as cell divisions per cell and day, the value is divided by the incubation period in days. For all drug treatments, cell division rates representing the average number of cell divisions per cell and day were calculated based on the cell division distribution (Fig. 2D). Cell division rates showed a significant decrease across all drug treatment conditions as measured by the *CeDaD* assay. However, this calculation only considers cell division when analyzing population growth, without accounting for cell death.

In order to assess the cell death rate within a population, a double staining approach using *Apotracker Green* and PI, derived from the commonly used annexin V/PI combination, was employed. Figure 2E, F shows representative analyses after 10^−6^ M volasertib and DMSO control treatment. After gating single cells, four subgroups were identified: viable cells (*Apotracker* negative, PI negative), apoptotic cells (*Apotracker* positive, PI negative), dead cells (*Apotracker* positive, PI positive), and a fourth category of artifacts (*Apotracker* negative, PI positive). This artifact population likely represents cell fragments or cells that died between *Apotracker Green* and PI staining as *Apotracker Green* and similar dyes would be able to enter and stain a dead cell through the ruptured cell membrane as does PI [47, 64]. This population, therefore, was excluded from further calculations. The cell death rate per day was calculated by relating apoptotic and dead cells to the total of all cells (viable, apoptotic, and dead cells). The inverse of this rate is the cell viability rate, with the sum of both always equaling one. This calculation assumes that cells are detectable for approximately 1 day between *Apotracker Green* detection of apoptosis and cell lysis. Available data, though limited, suggest similar durations [65, 66]. In HCT116 cells, treatment with 10^−6.5^ M YKL-5-124 and 10^−7.5^ M to 10^−6^ M volasertib significantly increased the cell death rate 2 days post-treatment, reducing viability (Fig. 2G). AMG 232, however, did not induce cell death in HCT116 cells. Of note, for dead cells it is not possible to distinguish between cells post apoptosis or necrosis, as annexin V-derived staining can stain dead cells due to their ruptured cell membrane [47, 64]. Consequently, we do not claim that the *CeDaD* assay can reliably distinguish between different forms of cell death. For a closer characterization of the cell death subtypes additional assay systems should be employed. Moreover, to evaluate the cell death rate of a cell population the combination of annexin V-derived staining and PI staining can theoretically be replaced by any other live/dead staining suitable for flow cytometry using the appropriate filters (green and red) such as a combination of Calcein AM and ethidium homodimer-1 double staining [67].

The analysis of cell division and cell death rates is a valuable approach to compare the effects of compounds on different cell lines. In our study, we employed the HCT116 cell model system, which includes cells with deletions in the *LIN37* and the *RB* genes, to investigate the impact of volasertib on cell division and viability [18]. LIN37 is a component of the DREAM transcriptional repressor complex, which, like RB, plays a role in cell cycle-dependent transcription and cell cycle control. *LIN37* knockout results in the loss of DREAM repressor function [68].

We compared the cell division rates (Fig. 2H) and cell death rates (Fig. 2I) between wild-type HCT116 and HCT116 LIN37^−/−^/RB^−/−^ cells following treatment with volasertib at concentrations of 10^−7.5^ M and 10^−6^ M. Notably, both concentrations similarly reduced cell division rates across both cell lines. However, treatment with 10^−6^ M volasertib resulted in a significantly lower cell death rate in HCT116 LIN37^−/−^/RB^−/−^ cells compared to wild-type cells. This suggests that the PLK1 inhibitor volasertib exerts a reduced effect on cell death induction in LIN37/DREAM- and RB-deficient cells compared to wild-type cells.

This observation highlights the utility of the *CeDaD* assay in providing insights into the mechanisms underlying differential cellular responses to diverse conditions. Without detailed data on the balance between cell cycle arrest and cell death induction, differences in proliferation patterns might be incorrectly attributed to variations in cell division activity alone. Given that the loss of LIN37 and RB disrupts the repression of cell cycle genes, leading to their deregulation [18], one may mistakenly conclude, based solely on *WST* assay data, that disparities in cell cycle arrest were responsible for the observed differences between the cell lines.

Importantly, this differential analysis of cell division and cell death activity was achievable from a single-cell population, starting with as few as 5 × 10^5^ initial cells, even under conditions that further reduced the cell population.

### Cell division and cell death rates adequately predict cell population growth

**Fig. 3:**
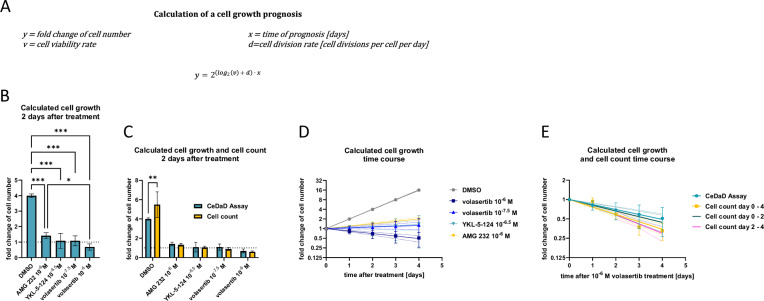
Cell division and cell death rates adequately predict cell growth behavior measured by cell count. Cell division and cell death rates calculated for HCT116 wt cells treated with AMG 232, YKL-5-124, or volasertib for 2 days were used to calculate a growth prognosis using the appropriate exponential growth equation (**A**). Calculated fold changes of cell number after 2 days of treatment were compared with each other (**B**, *n* = 3) and with cell count data (**C**, *n* ≥3). The growth behavior can also be described as a process by the exponential growth curve and the calculated fold changes of the cell number per day (**D**, *n* = 3). The calculated cell growth after treatment with 10^–6^ M volasertib was compared with cell count over 4 days after treatment (**E**, *n* ≥3). Exponential growth curves of the *CeDaD* assay data (blue line) as well as the cell counting data from day 0 to 4 (yellow line), the timeframe of the *CeDaD* assay (day 0 to day 2, dark-green line), and the timeframe after *CeDaD* measurement (day 2 to day 4, pink line) were generated. All exponential growth curves were calculated by least square regression and plotted with 95% confidence interval. Mean ± SD are given, and *P* values were calculated by two-way ANOVA (*P <0.05; **P <0.01; ***P <0.001).

Since cell division and cell death are the two key parameters that determine the growth potential of a cell population, they can be used to model an exponential growth curve (Fig. 3A). Generally, a cell division rate of one results in a doubling of the cell number, while a division rate of two leads to a fourfold increase. Conversely, a cell death rate of 0.5 results in a halving of the cell number, and a death rate of 0.75 causes a fourfold decrease.

For a straightforward calculation of the net change in cell population size due to both cell division and death, it is crucial that the parameters producing equal effects have the same absolute values but opposite signs—positive for the cell division parameter and negative for the cell death parameter. Therefore, if a cell division rate of one leads to a doubling of the cell population, then a cell death rate factor of minus one should correspondingly cause the population to reduce to half its size.

To accurately derive the cell death rate factor, the cell viability rate—which serves as the opposing factor to the cell death rate—is used as the logarithmic base two. This approach ensures that both processes are quantified in a manner that reflects their inverse relationship in influencing cell population dynamics. Importantly, the sum of the cell viability rate and cell death rate of a cell population is always one, and the cell viability rate is defined as a value between zero and one. Therefore, the result of the logarithm will always be negative, with smaller viability rates producing larger negative logarithmic values, while the cell division rate remains positive. This approach ensures that both parameters, which generate opposing effects of equal magnitude, have matching absolute values but opposite signs.

When calculating the fold change in cell number within a population over a given number of days, the exponential growth based on a factor of two is defined by the sum of the cell division rate and the logarithm base two of the cell viability rate, multiplied by the cultivation time in days. This formulation allows for a coherent integration of both cell division and death dynamics in predicting changes in cell population size.

Using the *CeDaD* assay to calculate the fold change in cell number 2 days after various test treatments, all treatments resulted in a significant reduction in population growth (Fig. 3B). Notably, this approach allowed us to detect significant differences between treatments, specifically between 10^−6^ M AMG 232 and 10^−6^ M volasertib, demonstrating the assay’s sensitivity in distinguishing the differential effects of these compounds on cell population dynamics. As discussed earlier, this significant difference is most likely due to the additional cytotoxic effects of volasertib rather than differences in cell cycle arrest (Fig. 2C, G). To validate whether these calculations accurately reflect the actual cell population growth over the 2-day measurement period, the factors determined by the *CeDaD* assay were compared to direct cell counts (Fig. 3C). Although the growth of an unimpaired cell population appears to be slightly underestimated, there were no significant differences between the calculated population growth from the *CeDaD* assay and the measured fold change in cell numbers obtained through cell counting under any of the drug treatment conditions.

The differing results observed in the DMSO control suggest that, under conditions of high proliferation and low apoptosis, the *CeDaD* assay may slightly underestimate the population growth. This underestimation could be attributed to false positives in the *Apotracker Green*/PI staining. Previous studies have indicated a slight overestimation of cell death by this assay, potentially due to membrane damage occurring during sample preparation [69, 70]. Obviously, technical artifacts which increase the apoptotic background have a more pronounced effect under conditions of low cell death, while they have minimal impact in scenarios with high cell death. Overall, these results strongly support the functionality of the *CeDaD* assay, particularly for analyzing impaired cell division and induced cell death.

### The *CeDaD* assay enables extrapolation of cell population growth

The *CeDaD* assay’s final application involves projecting cell population growth beyond the initial measurement period, as shown in the growth prognosis (Fig. 3D). Notably, the uncertainty of the prognosis inherently increases with the length of the projected cultivation time. Although this prognosis mirrors the trends observed in Fig. 3A, comparing these projected values with repeated cell counts can particularly reveal secondary treatment effects. An illustrative example of this comparison is provided for the 10^−6 ^M volasertib treatment (Fig. 3E).

The *CeDaD* assay prognosis (Fig. 3E, blue line) was compared with exponential growth regressions derived from cell counts over 4 days post-treatment (Fig. 3E, yellow line), from day 0 to day 2 (Fig. 3E, dark-green line), and from day 2 to day 4 (Fig. 3E, pink line). When comparing the complete data sets, the regression based on cell counts appears to slightly diverge from the *CeDaD* assay prognosis. However, when the regression is limited to the same 2-day period as the *CeDaD* assay measurements (Fig. 3E, dark-green line), the growth curves align much more closely.

Interestingly, the increase in the cell count curve from day 2 to day 4 is significantly lower than both the *CeDaD* assay curve and the initial 2-day cell count regression, suggesting secondary cytotoxic effects due to prolonged volasertib exposure. Similar effects were observed following treatments with 10^−6^ M AMG 232 and 10^−6.5^ M YKL-5-124, but not with 10^−7.5^ M volasertib (Supplementary Fig. 1). Importantly, no additional effects were detected in DMSO control-treated cells, as secondary effects are not expected under unimpaired growth conditions (Supplementary Fig. 1).

These drug-specific and concentration-dependent secondary effects would be challenging to discern solely from cell count data, as distinguishing these trends from random fluctuations can be difficult. The *CeDaD* assay growth prognosis, however, allowed us to identify secondary treatment effects that might otherwise have been obscured.

### Conclusion

The *CeDaD* assay is a valuable tool to simultaneously evaluate cell division activity and cell death induction within a single, small-scale cell culture population with minimal effort. Its ability to generate exponential growth curves for cell populations under investigation makes it especially beneficial when sample size is limited. Moreover, the *CeDaD* assay not only elucidates distinct mechanisms constraining population growth but also serves as a complementary tool to cell counting, enabling the visualization of secondary effects arising from treatments or variations in cultivation conditions.

## Materials and methods

### Cell culture and cell count analysis

Human colorectal carcinoma HCT116 wild-type (wt) cells (provided by Bert Vogelstein) and HCT116 LIN37^−/−^/RB^−/−^ cells were cultivated in Dulbecco’s modified Eagle’s medium (DMEM; Capricorn scientific). A detailed description of the HCT116 LIN37^−/−^/RB^−/−^ cell line was published earlier [18]. For standard growth medium, DMEM was supplemented with 5% fetal calf serum (FBS Good; PAN Biotech), 5% serum substitute (Panexin NTA, PAN Biotech), and 1% penicillin/streptomycin (PAN Biotech). All cell lines were cultivated under standard growth conditions with 37 °C and 10% CO_2_. For all treatment analyses aside from *WST* assay 5 × 10^5^ cells were seeded in 2 ml standard growth medium in six-well plates. One day after seeding, 500 µL standard growth medium containing 5x concentrated DMSO (1:200) with or without dissolved compounds, namely AMG 232 (MedChemExpress, HY-12296), YKL-5-124 (MedChemExpress, HY-101257), and volasertib (MedChemExpress, HY-12137) of varying concentrations was added. Cells cultured without compounds were split and reseeded on day 2 after the start of treatment. Again, 5 × 10^5^ cells were seeded in 2 ml standard growth medium in six-well plates, and the splitting factor was considered for calculations. The growth medium containing DMSO with compounds was changed after 2 days of incubation. For cell count analysis, cells were trypsinized, stained with trypan blue, and collected in a defined volume of standard growth medium and counted twice with the Countstar BioTech module (Countstar). PCR-based tests for mycoplasma contamination were performed using the Mycoplasma PCR Detection Kit (Applied Biological Materials).

### Cell death and division assay

For combined analysis of cell division and cell death via flow cytometry, cells were washed once with PBS and stained with 5 µM *CellTrace Violet* (Thermo Fisher) in 1 ml PBS based on a 6.6 mM *CellTrace Violet* solution in DMSO for 30 min. Then, the staining solution was removed and replaced by 2 ml standard growth medium with DMSO (1:1000) with or without dissolved compounds. Cells were treated with different compounds for 48 h. For staining of apoptotic and dead cells, the supernatant of the cell culture was collected, cells were trypsinized, added to the supernatant, and centrifuged at 500 × *g* for 5 min. An equal number of cells were resuspended in PBS containing 400 nM *Apotracker Green* (Biolegend), incubated for 30 min, and washed once with PBS. Before flow cytometry, PI (Sigma-Aldrich) was added to a final concentration of 0.1 ng/µl. Combined live cell staining of *CellTrace Violet* (laser: 405 nm, filter: 450/40 nm), PI (laser: 561 nm, filter: 585/15 nm), and *Apotracker Green* (laser: 488 nm, filter: 530/30 nm, mirror: 505 nm low pass) was analyzed by flow cytometry (LSRFortessa Cell Analyzer, Becton Dickinson). 10,000 events were recorded, and FlowJo Version 10 (Becton Dickinson) was used for data analysis. Single cells were gated from the whole cell population based on forward and side scatter analysis.

### *WST* assay

For the *WST-1* assay (Abcam), HCT116 cells were seeded in 96-well plates with 5 × 10^3^ cells per well in 80 µl standard growth medium. After 1 day, 20 µl of a 5× concentrated compound solution was added to each well containing DMSO for a final dilution of 1:1000. Two days after treatment, the *WST-1* assay was performed according to the manufacturer’s protocol. Specific absorption was measured at *λ* = 450 nm and as a reference at *λ* = 620 nm. As a blank value 100 µl standard growth medium with 10 µl *WST-1* reagent was used.

A detailed list of all used reagents and tools can be found in Supplementary File 1.

## Supplementary information

Supplemental File 1

Supplemental Figure 1

## Data Availability

Original data are available upon request.
