# Supplementary material for: CeDaD—a novel assay for simultaneous tracking of cell death and division in a single population
Source: Cell Death Discov. 2025 Mar 4;11:86. doi: 10.1038/s41420-025-02370-7 (PMC11880512; doi:10.1038/s41420-025-02370-7)
Supplement: Supplementary file 1 — Supplemental File 1 [file 41420_2025_2370_MOESM1_ESM.docx]

**Reagents and Tools Table**

| **Reagent/Resource** | **Reference or Source** | **Identifier or**  **Catalog Number** |
| --- | --- | --- |
| **Experimental Models** |  |  |
| HCT-116 cells *(H. sapiens)* | Bert Vogelstein | N/A |
| HCT-116 LIN37^-/-^/RB^-/-^ cells *(H. sapiens)* | Uxa et al., 2019 | Prof. Dr. Kurt Engeland, University Leipzig, Germany |
| **Chemicals, Enzymes and other reagents** |  |  |
| AMG 232 | MedChemExpress | HY-12296 |
| Apotracker Green | Biolegend | 427403 |
| CellTrace Violet | Thermo Fisher | C34557 |
| DMEM High Glucose (4.5 g/l), with L-Glutamine, with Sodium Pyruvate | Capricorn scientific | DMEM-HPA |
| DMSO | Serva | 20385 |
| Fetal bovine serum (FBS) | PAN Biotech | P40-37500 |
| Mycoplasma PCR detection kit | Applied Biological Materials | G238 |
| Panexin NTA Serum Substitute | PAN Biotech | P04-95750 |
| PBS | Biochrome/Merck | L 182-50 |
| Penicillin/streptomycin | PAN Biotech | P06-07100 |
| Propidium iodide | Sigma-Aldrich | P4864 |
| Trypan Blue | Sigma-Aldrich | T8154 |
| Trypsin-EDTA in DPBS (10x) | Capricorn scientific | TRY-1B10 |
| Volasertib | MedChemExpress | HY-12137 |
| WST-1 | Abcam | ab65473 |
| YKL-5-124 | MedChemExpress | HY-101257 |
| **Software** |  |  |
| FlowJo v10 | Becton Dickinson |  |
| GraphPad Prism v10 | GraphPad |  |
| **Other** |  |  |
| Countstar BioTech module | Countstar |  |
| LSRFortessa Cell Analyzer | Becton Dickinson |  |
| 800 TS microplate reader | BioTek |  |
