# Supplementary material for: CeDaD—a novel assay for simultaneous tracking of cell death and division in a single population
Source: Cell Death Discov. 2025 Mar 4;11:86. doi: 10.1038/s41420-025-02370-7 (PMC11880512; doi:10.1038/s41420-025-02370-7)
Supplement: Supplementary file 2 — Supplemental Figure 1 [file 41420_2025_2370_MOESM2_ESM.pptx]

## Slide 1
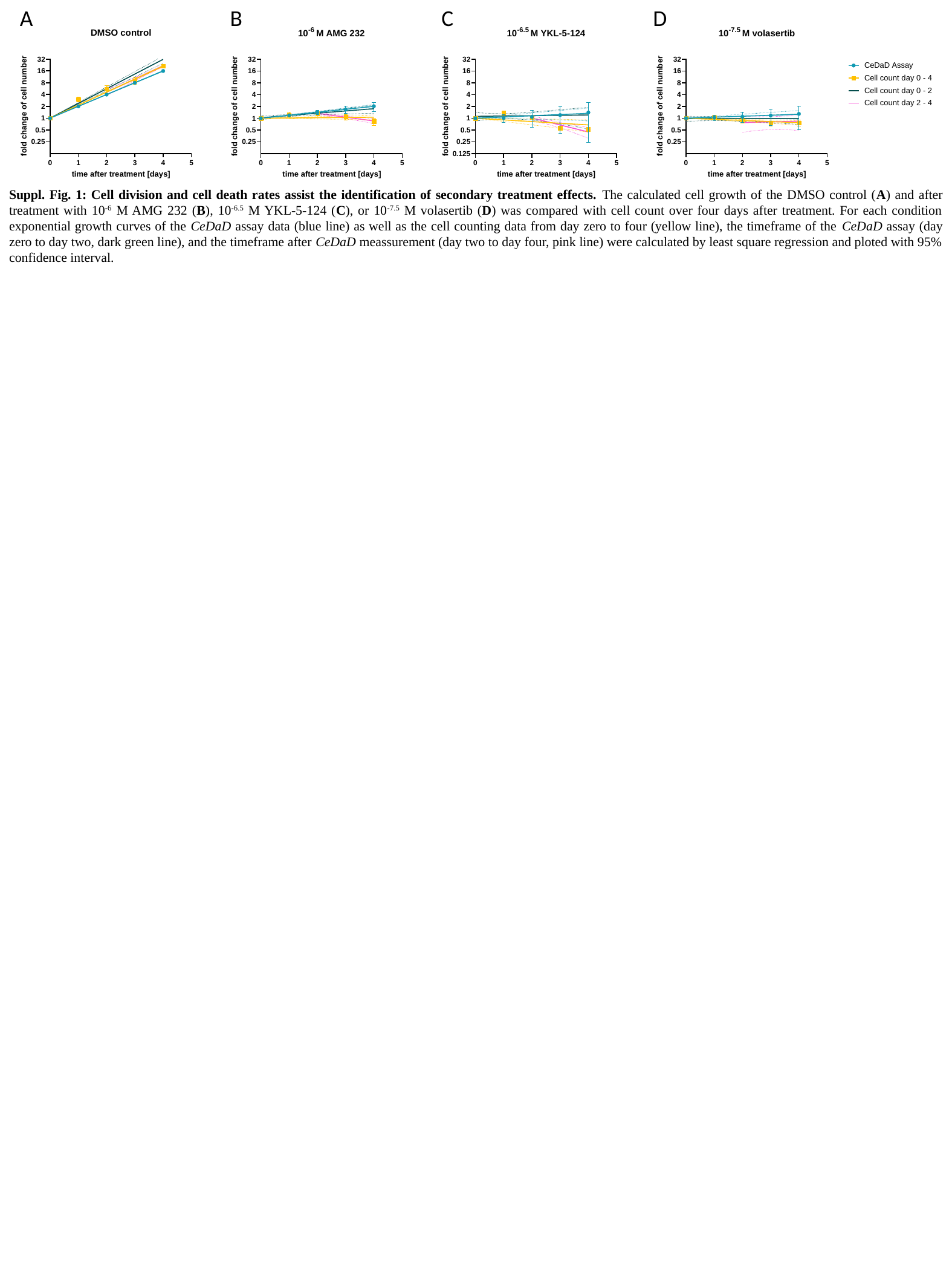

A
B
C
D
Suppl. Fig. 1: Cell division and cell death rates assist the identification of secondary treatment effects. The calculated cell growth of the DMSO control (A) and after treatment with 10-6 M AMG 232 (B), 10-6.5 M YKL-5-124 (C), or 10-7.5 M volasertib (D) was compared with cell count over four days after treatment. For each condition exponential growth curves of the CeDaD assay data (blue line) as well as the cell counting data from day zero to four (yellow line), the timeframe of the CeDaD assay (day zero to day two, dark green line), and the timeframe after CeDaD meassurement (day two to day four, pink line) were calculated by least square regression and ploted with 95% confidence interval.
